# Zika Virus Infects, Activates, and Crosses Brain Microvascular Endothelial Cells, without Barrier Disruption

**DOI:** 10.3389/fmicb.2017.02557

**Published:** 2017-12-22

**Authors:** Michelle P. Papa, Lana M. Meuren, Sharton V. A. Coelho, Carolina G. de Oliveira Lucas, Yasmin M. Mustafá, Flavio Lemos Matassoli, Paola P. Silveira, Paula S. Frost, Paula Pezzuto, Milene R. Ribeiro, Amilcar Tanuri, Mauricio L. Nogueira, Loraine Campanati, Marcelo T. Bozza, Heitor A. Paula Neto, Pedro M. Pimentel-Coelho, Claudia P. Figueiredo, Renato S. de Aguiar, Luciana B. de Arruda

**Affiliations:** ^1^Departamento de Virologia, Instituto de Microbiologia Paulo de Góes, Universidade Federal do Rio de Janeiro, Rio de Janeiro, Brazil; ^2^Departamento de Imunologia, Instituto de Microbiologia Paulo de Góes, Universidade Federal do Rio de Janeiro, Rio de Janeiro, Brazil; ^3^Departamento de Genética, Instituto de Biologia, Universidade Federal do Rio de Janeiro, Rio de Janeiro, Brazil; ^4^Núcleo de Neurociências da Faculdade de Farmácia, Universidade Federal do Rio de Janeiro, Rio de Janeiro, Brazil; ^5^Laboratório de Pesquisas em Virologia, Faculdade de Medicina de São José do Rio Preto, São José do Rio Preto, Brazil; ^6^Instituto de Ciências Biomédicas, Universidade Federal do Rio de Janeiro, Rio de Janeiro, Brazil; ^7^Laboratório de Alvos Moleculares, Departamento de Biotecnologia Farmacêutica, Faculdade de Farmácia, Universidade Federal do Rio de Janeiro, Rio de Janeiro, Brazil; ^8^Instituto de Biofísica Carlos Chagas Filho, Universidade Federal do Rio de Janeiro, Rio de Janeiro, Brazil

**Keywords:** Zika virus, endothelial cells, blood brain barrier, transcytosis, mouse experimental model

## Abstract

Zika virus (ZIKV) has been associated to central nervous system (CNS) harm, and virus was detected in the brain and cerebrospinal fluids of microcephaly and meningoencephalitis cases. However, the mechanism by which the virus reaches the CNS is unclear. Here, we addressed the effects of ZIKV replication in human brain microvascular endothelial cells (HBMECs), as an *in vitro* model of blood brain barrier (BBB), and evaluated virus extravasation and BBB integrity in an *in vivo* mouse experimental model. HBMECs were productively infected by African and Brazilian ZIKV strains (ZIKV_MR766_ and ZIKV_PE243_), which induce increased production of type I and type III IFN, inflammatory cytokines and chemokines. Infection with ZIKV_MR766_ promoted earlier cellular death, in comparison to ZIKV_PE243_, but infection with either strain did not result in enhanced endothelial permeability. Despite the maintenance of endothelial integrity, infectious virus particles crossed the monolayer by endocytosis/exocytosis-dependent replication pathway or by transcytosis. Remarkably, both viruses' strains infected IFNAR deficient mice, with high viral load being detected in the brains, without BBB disruption, which was only detected at later time points after infection. These data suggest that ZIKV infects and activates endothelial cells, and might reach the CNS through basolateral release, transcytosis or transinfection processes. These findings further improve the current knowledge regarding ZIKV dissemination pathways.

## Introduction

Zika virus (ZIKV) is an arthropod-borne virus (arbovirus), from *Flaviviridae* family, genus Flavivirus, which is now associated with a large spectrum of clinical manifestations and different forms of transmission, being a unique Arbovirus (WHO, 2016). ZIKV was first isolated in 1947, in Uganda, during a surveillance study in mosquitoes and primates (Dick et al., 1952). Until 2007, few sporadic human cases had been reported in African and Southeast Asiatic countries, and were associated to mild clinical manifestations. After human-associated ZIKV outbreaks in Micronesia and Pacific Islands in 2007 and 2013, the virus spread to the Americas, and has now been reported in more than 60 countries around the world (Lanciotti et al., 2008; Cao-Lormeau et al., 2014; Faria et al., 2016; WHO, 2016).

ZIKV circulation positively correlated with enormous increase in the number of cases of microcephaly, suggesting a causal association between ZIKV infection during pregnancy and neonatal microcephaly (Calvet et al., 2016; Oliveira Melo et al., 2016; Rasmussen et al., 2016; Schuler-Faccini et al., 2016). This was further supported by virus detection in the brains and in amniotic fluid of fetuses with microcephaly by qRT-PCR, immunohistochemistry and electron microscopy (Calvet et al., 2016; Martines et al., 2016; Mlakar et al., 2016). Later on, ZIKV congenital transmission was associated to further fetal malformations, including several neurological manifestations, which are now described as congenital Zika syndrome (CZS) (Miranda-Filho et al., 2016; Lucey et al., 2017). Meningoencephalitis was also reported in ZIKV-infected adult patients and during experimental infection of rhesus monkeys, and the viral RNA was detected in their cerebrospinal fluid (Carteaux et al., 2016; Dudley et al., 2016). These lines of evidence suggest that virus replication may be, eventually, associated to invasion of central nervous system (CNS).

In spite of multiple sources of evidence indicating that ZIKV penetrates CNS, the mechanism associated to this infiltration is unknown. Neurotropic viruses may access the brain by either neural or hematogenic pathways; in the latter case, viruses or virus-infected cells must cross the blood brain barrier (BBB) (Salinas et al., 2010; Luethy et al., 2016). BBB is a dynamic structure of specialized cells that limits the passage of circulating molecules and cells to the brain, and restricts the entry of pathogens, including viruses or virus-infected cells to the CNS. Brain microvascular endothelial cells are the most prominent cell type, responsible for this control, acting in concert with astrocytes, microglia, pericytes, and neurons (Ballabh et al., 2004; Miner and Diamond, 2016). Disruption of the BBB, commonly due to increased endothelial cell permeability, is a hallmark of CNS infections and can be induced by virus replication or neuroinflammation (Daniels and Klein, 2015). Alternatively, it was suggested that increased BBB permeability might not be essential for lethal disease induced by another flavivirus infection model (Morrey et al., 2008). Infection and activation of endothelial cells in the retina and placenta of ZIKA experimental models have been previously observed (Noronha et al., 2016; Singh et al., 2017; Vermillion et al., 2017); however, the role of the virus replication in endothelial cell for virus dissemination or extravasation to other tissues has not been addressed.

Two different ZIKV lineages have been epidemiologically characterized, named African and Asian, the latter being associated to the epidemic outbreak in Brazil (Calvet et al., 2016; Weaver et al., 2016; Zhu et al., 2016). It was recently demonstrated that ZIKV infects human brain microvascular endothelial cells, which is a model of BBB tissue (Bayer et al., 2016; Mladinich et al., 2017). HBMECs infection by ZIKV was associated to a persistent infection with no evidences of increased permeability *in vitro* (Mladinich et al., 2017). Nevertheless, the effect of different ZIKV strains, obtained from mosquitoes and mammal cells, in HBMECs survival, activation and permeability worth to be further addressed and compared. Importantly, it is still not established whether a systemic infection with ZIKV *in vivo* may promote virus extravasation and infection of CNS. Here, we described that different strains of ZIKV productively infect HBMECs and induce cell activation, with increased production of type I and type III IFN, IL-6, and CCL5, confirming previous data obtained with a different strain (Mladinich et al., 2017). HBMEC infection with either virus did not result in significant disruption of the monolayer permeability. Still, infectious virus crossed the endothelial monolayer through replication and transcytosis dependent pathways. Remarkably, infection of A129 mice with either virus strain did not result in BBB permeability at early time points after infection, although high viral load had been detected in the brain. Subtle BBB alteration was detected at later time points post infection, which might be a result of a virus-induced inflammatory response. These data suggest that ZIKV virus cross BBB through transcytosis or transinfection after endothelial cell infection and activation.

## Materials and methods

### Ethical statements

Blood samples (buffy coats) from healthy donors were obtained anonymously from the Hemotherapy Service from the Hospital Universitário Clementino Fraga Filho (HUCFF) of Universidade Federal do Rio de Janeiro (UFRJ). The study protocol was approved by the Experimental Ethics Committee of UFRJ (Permit Number: 105/07) and the review board waived the need for informed patient consent.

A129 (deficient of IFNARI) mice were obtained from the mice facility of the Instituto de Microbiologia, Universidade Federal do Rio de Janeiro (IMPPG, UFRJ), Brazil. The animals were bred and housed according to institutional policies for animal care and usage and the protocol was approved by The Ethics Committee of Animal Care and Use (Comite de Etica no Uso de Animais-CEUA) from Centro de Ciencias da Saude, UFRJ (Permit Number: no104/16).

### Virus and cells

Vero cells were cultured in DMEM supplemented with L-glutamine and 5% fetal bovine serum (Life Technologies, Grand Island, NY) and maintained at 37°C with 5% CO_2_. C6/36 mosquito cell line were cultured at 28°C in Leibovitz (L-15) medium (Life Technologies) supplemented with 10% of tryptose phosphate broth, 0.75% sodium bicarbonate, 0.2% of L-glutamine (Sigma-Aldrich, St Louis, MO), and 10% FBS (Life Technologies). Human brain microvascular endothelial cells (HBMEC) have been previously described (Nikolskaia et al., 2006) and were kindly given by Dr. Julio Scharfstein (Instituto de Biofísica Carlos Chagas Filho, UFRJ). The cells were cultured in M199, supplemented with L-glutamine, non-essential aminoacids, and 10% FBS (Life Technologies). Peripheral blood mononuclear cells (PBMC) were obtained after centrifugation of buffy coats samples over ficoll-hypaque gradients and cultured with RPMI supplemented with L-glutamine, and 10% FBS.

ZIKV strain MR766 (ZIKV_MR766;_ ATCC VR1838) was propagated in Vero or C6/36 cells, as indicated in each experiment. ZIKV_PE243_ (gene bank ref. number KX197192) was isolated from a febrile case in the state of Pernambuco, Brazil, and was kindly given by Dr. Ernesto T.A. Marques Jr. (Centro de Pesquisas Aggeu Magalhães, FIOCRUZ, PE, Brazil; and Center for Vaccine Research, University of Pittsburgh, PA). Viruses were propagated in C6/36 cells and sequence analysis was performed after six passages. In some experiments, viruses were also propagated in Vero cells, as indicated in each experiment. ZIKV_BR−SP_ (gene bank ref. number KU497555; kindly given by Pedro Vasconcelos, Instituto Evandro Chagas, FIOCRUZ, PA, Brazil) was isolated from a mild ZIKV case, in the state of Paraíba, Brazil, and it was distributed as part of ZIKA FAPESP NETWORK after four passages in C6/36 cells (Faria et al., 2016). Viral titers were determined by plaque assay in Vero cells, as previously described (Coelho et al., 2017) and are indicated as PFU/ml. Supernatants of non-infected C6/36 or Vero cells cultured in the same conditions were used as mock controls. Inactivated virus (iZIKV) was obtained after U.V. exposition for 2 h and the inactivation was confirmed by qRT-PCR in Vero cells.

### HBMECs and PBMCs infection

HBMECs were infected with ZIKV_MR766_ or ZIKV_PE243_ with a MOI of 1 for 2 h, at 37°C in 5% CO_2_ atmosphere, for virus adsorption. As a control, the cells were incubated with supernatant of non-infected Vero or C6/36 cells (mock-infected). Cells were, then, washed with PBS and cultured with complete medium. After different time points, cells and supernatants were harvested and virus infection, cell survival, and cytokine secretion were evaluated as described below.

PBMCs were infected at the same conditions and, after 48 h, supernatants were harvested, and stored at −80°C. Supernatants from PBMC treated with mock, or infected with ZIKV_PE243_ or ZIKV_MR766_ were inactivated by U.V. radiation for 2 h and used as conditioned medium (iCM-PBMC). Virus inactivation was confirmed by qRT-PCR and plaque assay after 48 h infection of Vero cells.

### Analysis of HBMECs infection by immunofluorescence, qRT-PCR, and plaque assay

HBMECs were mock-treated or infected with ZIKV_PE243_ or ZIKV_MR766_ at different MOIs. After 48 hpi, cells were blocked, permeabilized, and stained with anti-flavivirus 4G2 antibody (ATCC HB112), followed by AlexaFluor488-conjugated anti-mouse IgG (Life Technologies). HBMEC infection was then analyzed by immunofluorescence, using OLYMPUS IX81 microscopy.

Viral replication was also analyzed by qRT-PCR. Cells were treated as described and, after different time points, cells, and supernatants were harvested and RNA was isolated using TRIZOL reagent (Life Technologies), according to the manufacturer's instructions. Treatment with DNAse I (Ambion, Thermo Fischer) was performed to prevent genomic DNA contamination and first strand cDNA was synthesized using High-Capacity cDNA Archive Kit (Applied Biosystems), according to the manufacturer's instructions. cDNAs were subjected to quantitative real-time PCR for detection of viral RNA using a StepOnePlus Real-time PCR system and Taqman Master Mix Reagents (Applied Biosystems), using primers and probe specific for protein E sequence, as previously described (Lanciotti et al., 2008). cDNA obtained from virus samples ranging from 75,000 to 0.75 PFU/ml were used to construct a standard curve for estimating the genome copy number of ZIKV (RNA equivalent).

To evaluate secretion of infectious viral particles by HBMECs, cells were infected as described and, after different time points, the supernatants were harvested and titrated by plaque assay, using Vero cell line.

### Cell viability assays

HBMECs were infected with ZIK_PE243_ or ZIKV_MR766_ propagated in C6/36 cells and determination of cell viability after different time points was carried out using XTT 2,3-Bis-(2-Methoxy-4-Nitro-5-Sulfophenyl)-2H-Tetrazolium-5-Carboxanilide (XTT) (Sigma-Aldrich, St. Louis, MO). Cells were incubated with XTT solution for 2–4 h and metabolization was evaluated by spectrophotometry at 450 nm OD. One percent Triton X100 was used as a positive control.

Annexin V and propidium iodide (AnnV/PI) staining was also performed in HBMECs-infected cells. Briefly, HBMECs were mock treated or infected with ZIKV_PE243_ or ZIKV_MR766_ at a MOI of 1. After different time points, cells were stained with FITC-AnnexinV and 2.5 μg/ml of PI and analyzed by flow cytometry. Alternatively, cells were incubated with Muse Annexin V and Dead cell kit (Millipore) and were also analyzed by flow cytometry. Samples acquisition and analysis were performed using FACSCanto equipment (BD Biosciences) and Flow Jo software. In addition, supernatants were harvested and release of lactate dehydrogenase was measured by LDH assay, according to manufacturer's protocol (Bioclin, RJ, Brazil).

Plasma membrane integrity and apoptosis induction were also evaluated by PI and TUNEL staining, respectively, and fluorescence microscopy analysis. HBMECs were seeded on transwell inserts (Costar—Corning®) and infected with ZIKV_PE243_ or ZIKV_MR766_, as described. After 72 hpi, we performed TUNEL staining (*In Situ* Cell Death Kit—Roche), following manufacturer's instructions, or the cells were incubated with Propidium Iodide (BD Biosciences). The fluorescence was evaluated using OLYMPUS IX81 microscopy. Staurosporin (50 μM; Sigma- Aldrich) and Triton X-100 (0.1%; J.T. Baker) were used as positive controls for TUNEL and PI staining, respectively.

### Evaluation of cytokine production

HBMECs were infected as described and, the indicated time points, supernatants were harvested and cytokine concentrations were measured using a human cytokine 27-plex multiplex assay (Bio-Plex kit) and Bio-Plex® MAGPIX™ Multiplex equipment (Bio-Rad). The secretion of IL-6, IL-8, CCL5, and CXCL10 were confirmed by ELISA, according to manufacturer's protocol (Peprotech).

IFN-β, IFN-λ1, and IFN-λ4 expression in the cell lysates were measured at different time points post infection by qRT-PCR, and GAPDH expression was measured as a housekeeping control gene. RNA was extracted using TRIZOL reagent (Life Technologies), and first strand cDNA was synthesized using High-Capacity cDNA Archive Kit (Applied Biosystems), according to the manufacturer's instructions. Expression of type I and type III IFN mRNAs were measured using SYBR Green (Applied Biosystems), using the following primers: IFN-β sense: 5′-TAG CAC TGG CTG GAA TGA GA-3′, IFN-β antisense 5′-TCC TTG GCC TTC AGG TAA TG-3′; IFN-λ1 sense: 5′-GGG AAG CAG TTG CGA TTT AG-3′ and IFN-λ1 antisense 5′-GAT TTG AAC CTG CCA ATG TG-3′; IFN-λ4 sense: 5′-AGG GTC CTT AAC CGA CTG TG-3′ and IFN-λ4 antisense 5′-AAA CAA CCA ATG CGA TCA AA-3′; GAPDH sense 5′-GTG GAC CTG ACC TGC CGT CT-3′ and GAPDH antisense 5′-GGA GGA GTG GGT GTC GCT GT-3′. All qRT-PCR were performed with a standard PCR: the samples were subjected to 50°C for 2 min, 95°C for 10 min and 40 cycles of denaturation (95°C, 15 s), primer annealing (55°C, 30 s), and primer extension (60°C, 1 min). Next, the samples were subjected to a melt curve to eliminate primer dimers: 95°C, 15 s; 60°C, 1 min and 95°C, 15 s. Comparative CT method (ΔΔ Ct) was used to quantify gene expression levels with GAPDH used for normalization. Results are expressed as Mean ± *SD*. Kruskal–Wallis test One-way ANOVA was employed to compare differences between expressions of target genes with a significance level of 0.05.

### Endothelial permeability assay

HBMECs (5 × 10^4^ cells/well) were seed onto transwell inserts (Corning Costar, ME, USA; 0.4 μm membrane) and confluence was monitored everyday by measuring transendothelial electrical resistance (TEER) across cell monolayers using a Voltohmmeter (Millicell ERS-2). TEER was calculated after subtracting the resistance value in each experimental situation by the blank resistance of the membrane (without cells), and considering that resistance is inversely proportional to the area of the membrane. Resistance values were reported as “Ω/cm^2^” and the experiments were performed when a high resistance (>80 Ω/cm^2^) was reached (Mahad et al., 2006; Srinivasan et al., 2015). Cells were infected, from the apical side, with ZIKV_PE243_ or ZIKV_MR766_ (propagated in C6/36 cells) with a MOI of 1. As negative controls, cells were cultured with mock supernatants obtained from C6/36 cells. Staurosporin (STS; 10 μM; Sigma-Aldrich) was used as positive control. In some experiments, after virus adsorption, the cells were treated with Chloroquine diphosphate (50 μM; kindly supplied by FarManguinhos, Fiocruz, Rio de Janeiro, Brazil), or Nystatin (10 μM; Sigma-Aldrich), or Brefeldin A (BFA; 2 μg/ml; eBiosciences, San Diego, CA). After 72 h, supernatants were harvested and cells were incubated with FITC-conjugated BSA for 30 min. BSA extravasation was evaluated by measuring fluorescence intensity in the lower chamber, using spectrofotometer SpectraMax i3 (Molecular Devices, Lagerhausstrasse, Austria). The Permeability Coefficient (Pd) of albumin was calculated as: Pd = [A]/t × 1/A × V/[L], where [A] is the albumin concentration in lower chamber, t refers to time in seconds, A indicates the area of the membrane (in cm^2^), V is the volume of the bottom chamber, [L] is the albumin concentration in upper chamber. Data was normalized in relation to cells culture in culture medium only.

Virus RNA was measured in the upper and lower chamber of the transwell by qRT-PCR, as previously described. Also, the medium harvested from the lower chamber was inoculated into a Vero cell culture and virus RNA in the cell lysates and supernatants were measured by qRT-PCR. To confirm the presence of infectious virus in the abluminal chamber, Vero cells (8 × 10^4^ cells/well) were seeded in the lower chamber and, at 48 hpi, a real time PCR targeting viral negative strand was performed with the cell lysates. Briefly, intracellular viral RNA was extracted as described, and reverse transcription was performed using 835 forward primer instead of random primers (Lanciotti et al., 2008). Real time PCR was performed using TaqMan Universal Master Mix, as described.

In some experiments, HBMECs were also cultured with 50% conditioned medium obtained from PBMCs mock-treated or infected with ZIKV_PE243_ or ZIKV_MR766_ (iCM-PBMC) and cell permeability was evaluated as described.

### Analysis of endothelial cell adhesion protein by immunofluorescence

HBMECs were cultured onto coverslips and cells were mock treated or infected as described. After 72 hpi, cells were fixed with 4% paraformaldehyde in PBS for 20 min, washed in PBS twice and permeabilized with 0.1% Triton X-100 (Sigma Aldrich) plus 3% bovine serum albumine (BSA—Sigma Aldrich) for 25 min. Cells were incubated with both an anti-flavivirus 4G2 antibody (ATCC HB112) and anti-ß-catenin (Sigma Aldrich) antibodies diluted in PBS−3% BSA overnight at 4°C. After washing in PBS, cells were incubated with the AlexaFluor488-conjugated anti-mouse IgG (4G2 staining), or with AlexaFluor594-conjugated goat anti-rabbit IgG (β-catenin staining) (Life technologies) for 40 min. The cells were then washed three times in PBS and incubated with DAPI for 5 min. Following thorough washing with PBS, the coverslips were mounted with prolong gold antifade reagent (Life technologies) and imaged on a Zeiss LSM 710 confocal.

### Mouse infection

A129 mice (4 weeks age) were infected with 2 × 10^5^ PFU of ZIKV_PE243_ or ZIKV_MR766_ by i.v route. Mock supernatants were used as negative control. To evaluate virus replication, mice brains were removed at 2 or 5 days p.i. and macerated. RNA isolation, cDNA, and qRT-PCR were performed as described previously.

### Analysis of blood brain barrier integrity *in vivo* by evans blue staining

A129 mice were infected with ZIKV_PE243_ or ZIKV_MR766_, as described. Two or five days post infection, mice were i.v. injected with 0.5% Evans blue solution (EB, 200 μL per mouse) (Vetec, Rio de Janeiro, BR). After 1 h, mice were perfused with PBS and their brains were carefully removed. Brains were weighted, placed in 1 mL formamide (Vetec) and kept at room temperature for 3 days for stain extraction. As a positive control for BBB permeabilization, we used C57BL/6 mice infected with Plasmodium berguei ANKA, an experimental model of cerebral malaria (Reis et al., 2012). Mice were intraperitoneally inoculated with 10^5^ infected erythrocytes and BBB permeability was assayed 7 days after infection. The amount of Evans blue in solution was measured by optical spectroscopy using SpectraMax i3 (Molecular Devices), and calculated using a standard curve. Blood brain barrier permeability was estimated as μg Evans Blue/mg tissue.

### Immunoglobulin G staining

A129 mice were infected with ZIKV_PE243_ or ZIKV_MR766_, as described. Two or five days post infection, mice were deeply anesthetized and then transcardially perfused with ice-cold 0.9% saline, followed by 4% paraformaldehyde (PFA) in 0.1 M sodium phosphate buffer, pH 7.4. Brains were rapidly removed from skulls, postfixed in PFA for 1 d at 4°C, and cryoprotected in a PFA solution containing 20% (w/v) sucrose overnight. The frozen brains were then sectioned into 20 μm-thick coronal sections using a sliding microtome (Leica). Slices were collected in a cold cryoprotectant solution (0.05 M sodium phosphate buffer, pH 7.4, 30% ethylene glycol, 20% glycerol) and stored at −20°C. Free-floating sections were washed with 0.4% Triton X-100 in PBS (3 × 10 min) and then incubated for 30 min in a blocking solution containing 4% normal goat serum (Thermo Fisher Scientific) in PBS. Sections were washed with PBS (3 × 10 min), followed by an overnight incubation with a biotinylated goat anti-mouse IgG (H+L) antibody (1:500, Vector Laboratories). Binding was visualized using the peroxidase-based Vectastain ABC kit and 3,3′-diaminobenzidine (Vector Laboratories). Tissues were thereafter dehydrated through graded concentrations of alcohol, cleared in HistoChoice®Clearing agent (Sigma-Aldrich) and coverslipped with Organo/Limonene Mount™ (Sigma-Aldrich). Slides were scanned with a Pannoramic MIDI II scanner (3DHISTECH).

### Immunohistochemistry analysis of mice brain tissues

A129 mice were infected with ZIKV_PE243_, as described. Five days post infection, mice were transcardially perfused with cold phosphate-buffered saline (PBS) solution followed by fresh ice-cold 4% formaldehyde (PFA). The brain samples were fixed in 4% PFA, and cryoprotected with sucrose 30%. Slides with coronal frozen brain sections (30 μm-thick) were fixed in acetone for 30 min, washed twice with PBS and slides were blocked with PBS supplemented with 10% FBS and 1% NDS (normal donkey serum) (blocking buffer), for 1 h. The tissues were then incubated with mouse antibodies anti-4G2 antibody, or anti-VE-cadherin (2 μg/ml; Santa Cruz Biotechnology), or anti-occludin (2.5 μg/ml; Invitrogen), all diluted in blocking buffer. Primary antibodies were incubated overnight, at 4°C, in humid chamber. After washing in PBS, cells were incubated with Alexa Fluor 488-conjugated anti-mouse IgG diluted in blocking buffer (2.5 μg/ml; Invitrogen), for 1 h, at 4°C, in humid chamber. Then, the slides were mounted in Prolong Gold Antifade with DAPI (Invitrogen) and imaged on a Zeiss Axio Observer Z1 microscope equipped with an Apotome module.

### Statistical analysis

Data were analyzed using the GraphPad Prism software (GraphPad Software, San Diego, CA, USA). Comparisons among groups were performed by two way ANOVA; *p* < 0.05 were considered statistically significant.

## Results

### HBMECs are productively infected by ZIKV

**Figure 1:**
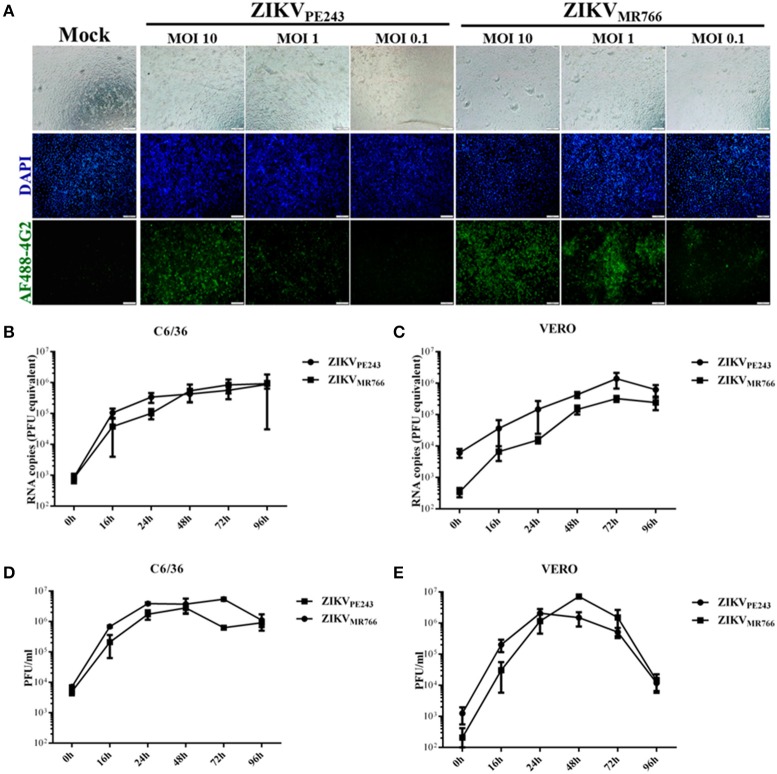
HBMECs are productively infected by ZIKV. **(A)** HBMECs were mock treated or infected with ZIKV_PE243_ or ZIKV_MR766_, at the indicated MOI. After 48 h, cells were stained with 4G2 antibody and analyzed by immunofluorescence. **(B,C)** HBMECs were infected with ZIKV_PE243_ or ZIKV_MR766_ (MOI 1), produced in C6/36 cells **(B)** or produced in Vero cells **(C)**. After different time points, supernatants were harvested and viral RNA was measured by qRT-PCR. **(D,E)** HBMECs were infected with ZIKV_PE243_ or ZIKV_MR766_ (MOI 1), produced in C6/36 cells **(D)** or produced in Vero cells **(E)**. After different time points, supernatants were harvested and infectious particles were titrated by plaque assay. Data are represented as mean ± *SD* of two independent experiments.

Human brain microvascular cells were previously shown to be susceptible to ZIKV (Bayer et al., 2016); however, a productive infection was not fully addressed. We further investigated HBMEC infection by different strategies. Initially, cells were infected with different MOIs of ZIKV_PE243_ or ZIKV_MR766_ and, after 48 h, ZIKV antigen expression was analyzed by immunofluorescence, using anti-flavivirus 4G2 antibody. We observed that virus antigen was clearly detected after infection with either ZIKV strain, with 10 or 1 MOI (Figure 1A). The replication efficiency of ZIKV_PE243_ and ZIKV_MR766_, obtained from C6/36 or Vero cell lines, were then compared by performing kinetic measurements of virus RNA and infectious particles release. We observed that infection with both strains resulted in similar level of virus RNA release, independent of the virus source (Figures 1B,C). Analysis of the production of infectious particles by plaque assay confirmed that HBMEC were permissive to infection with both ZIKV strains (Figures 1D,E). Infection with viruses produced in Vero cells resulted in slightly increased replication of ZIKV_MR766_; in addition, a significant drop in viral secretion was observed at 96 hpi (Figure 1E), which was not observed when cells were infected with viruses obtained from C6/36 cells (Figure 1D). Therefore, further investigation of the effects of ZIKV infection on HBMEC physiology was performed using C6/36-derived virus stocks.

### ZIKV_MR766_ induces higher cytotoxicity in HBMECs than ZIKV_PE243_

**Figure 2:**
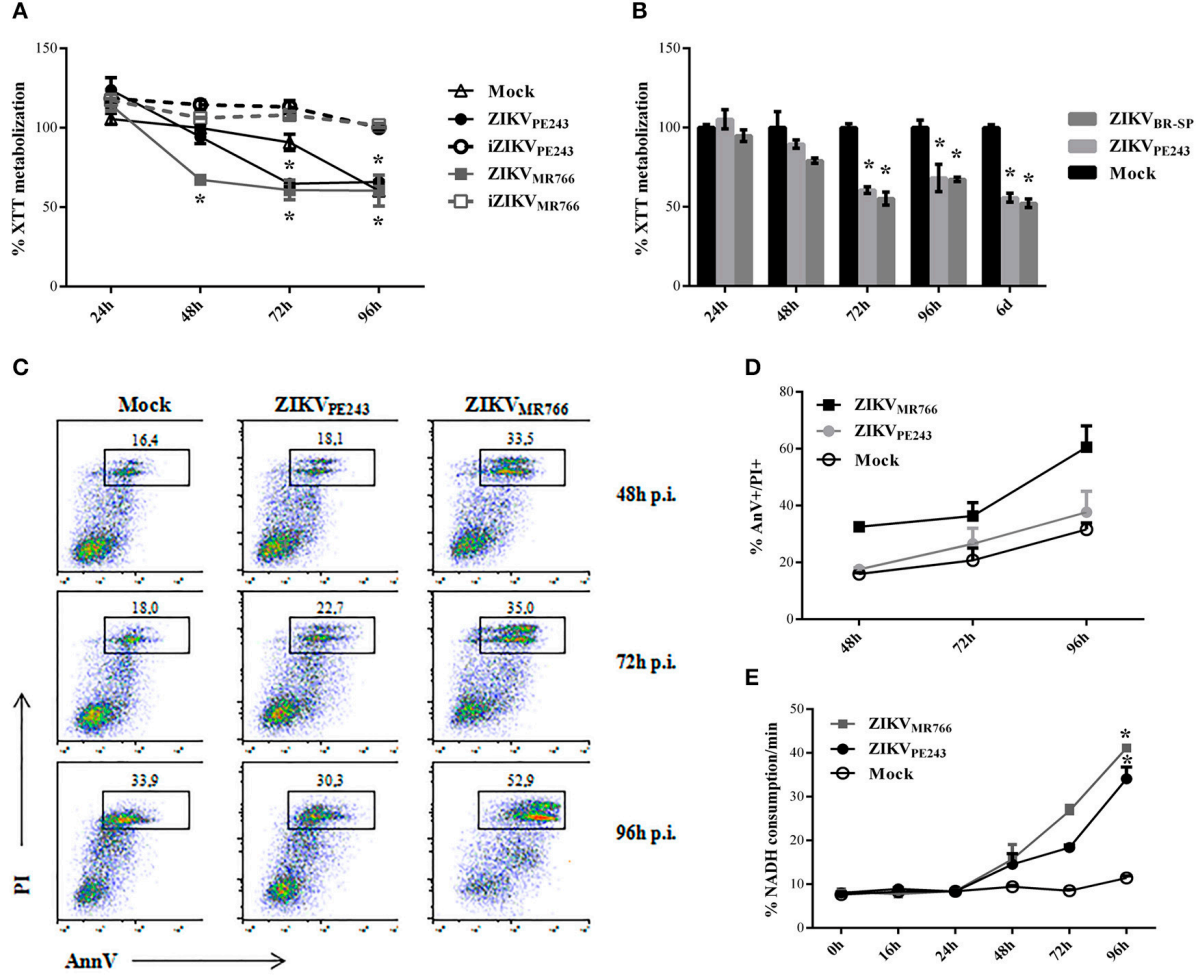
Cell viability of HBMECs infected with ZIKV. **(A)** HBMECs were mock-treated or cultured with ZIKV_PE243_ or ZIKV_MR766_, produced in C6/36 cells, in their native and inactivated forms (iZIKV). XTT metabolization was assayed at the indicated time points, and normalized according to the values obtained in cell cultures maintained in culture medium only. Data are represented as mean ± *SD* of six independent experiments. ^*^p < 0.05 in relation to mock. **(B)** HBMECs were mock-treated or infected with ZIKV_PE243_ or ZIKV_BR−SP_ and XTT assay was performed as in **(A)**. **(C,D)** HBMECs were infected with ZIKV_PE243_ or ZIKV_MR766_. Cells were stained with AlexaFluor488-Annexin V (AnnV) and propidium iodide (PI) and were evaluated by flow cytometry, at the indicated time points. **(C)** Dot plot indicating the percentage of AnV^+^PI^+^ cells at each time point, in a representative experiment. **(D)** Average percentage of AnnV^+^PI^+^cells at each time point from three independent experiments. **(E)** Culture supernatants were harvested and LDH release was evaluated by measurement of NADH consumption/min in a LDH activity assay. Data are represented as mean ± *SD* of three independent experiments. ^*^p < 0.05 in relation to mock.

We then evaluated whether infection with ZIKV would affect HBMEC physiology and survival. Initial analyses were performed by XTT metabolization assay at different time points post infection, normalized according to cell cultures with culture medium only. Infection with ZIKV_MR766_ resulted in decreased XTT metabolization levels in comparison to mock-treated cells, from 48 hpi and thereafter, suggesting that HBMEC infection with this strain induced cell death. On the other hand, alteration in XTT metabolization by infection with ZIKV_PE243_ was not detected until 72 hpi, indicating that the Brazilian strain was not associated to a severe cytophatic effect in these cells (Figure 2A). ZIKV-induced altered cell metabolism or viability depended on virus replication, since HBMEC culture with inactivated viruses did not result in any alteration of XTT metabolization. HBMECs cell death was also evaluated after infection with another Brazilian virus isolated (ZIKV_BR−SP_), and the results obtained were very similar to the ones obtained with ZIKV_PE243_ (Figure 2B).

Cell viability was also accessed by Annexin V/PI staining and LDH release. Flow cytometry analysis demonstrated that around 35% of cells infected with ZIKV_MR766_ were AnnV/PI-positive after 72 hpi, in contrast to 20% of the cells infected with the Brazilian strain. About 70% of HBMECs were still alive even at 96 h after ZIKV_PE243_ infection, when almost 60% of the cells infected with ZIKV_MR766_ were dead (Figures 2C,D). This kinetic was consistent with the detection of LDH release, which was observed from 48 hpi; and peaked at 96 hpi (Figure 2E). Similarly, infection with ZIKV_MR766_ resulted in higher levels of LDH activity detected in the supernatants, in comparison to infection with ZIKV_PE243_.

To confirm that infection with ZIKV_PE243_ was not associated to a remarkable cytopathic effect, HBMEC viability was also evaluated by different techniques, including flow cytometry analysis, based on 7AAD staining; fluorescence microscopy analysis, after PI and TUNEL staining; and cell counting with Trypan blue exclusion dye. Flow cytometry analysis demonstrated that dead cells could only be detected after 72 hpi with ZIKV_MR766_, but not in mock-treated or ZIKV_PE243_-infected cells (Supplementary Figure 1A). Fluorescence analysis did not indicate a significant TUNEL staining in any experimental situation, although PI staining could be detected in ZIKV infected cells (Supplementary Figure 1B). Finally, cell proliferation and viability was accessed by following the absolute cell numbers recovered after tripsinization of HBMECs infected with ZIKV_PE243_ or ZIKV_MR766_, in comparison to Vero cells. We observed that HBMECs infected with ZIKV_PE243_ were still able to replicate during 48 h culture, although the absolute number of cells recovered after 48 h was a little lower (about 7–12%) than the mock-treated cells (Supplementary Figure 1C). HBMECs infected with ZIKV_MR766_ also replicated, but at much lower efficiency. In contrast, the proportion of ZIKV-infected Vero cells recovered after 48 hpi was much lower (40–50%), in comparison to mock-treated cells (Supplementary Figure 1D). Taken together, these data corroborates with previous observation that HBMEC is more resistant to ZIKV-induced CPE than other cell types, such as Vero cells.

### ZIKV stimulates the secretion of proinflammatory cytokines by HBMECs

**Figure 3:**
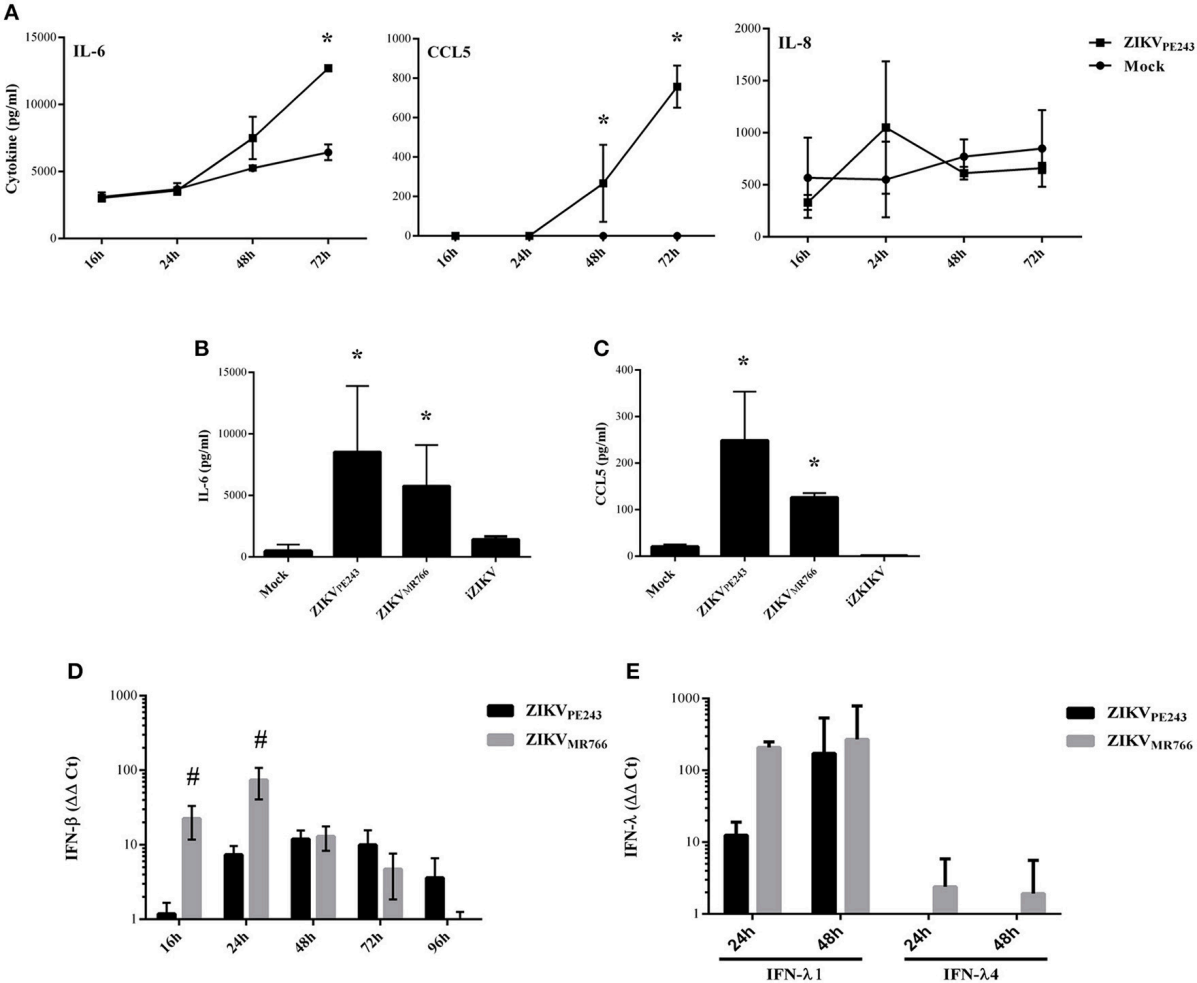
ZIKV induces the secretion of inflammatory cytokines and chemokines by HBMECs. **(A)** HBMECs were mock-treated or infected with ZIKV_PE243_, with a MOI of 1. After different time points, supernatants were harvested and IL-6, IL-8, and CCL5 were measured by ELISA. Data are represented as mean ± *SD* of two independent experiments and ^*^p < 0.05 in relation to mock. **(B,C)** HBMECs were mock-treated or infected with ZIKV_PE243_ or ZIKV_MR766_, with a MOI of 1. After 48 hpi, supernatants were harvested and IL-6 **(B)** and CCL5 **(C)** were measured by ELISA. Data are represented as mean ± *SD* of eight independent experiments and ^*^p < 0.05 in relation to mock. **(D,E)** HBMECs were treated as in **(B)** and cell lysates were obtained at the indicated time points. Expression of IFN-β mRNA **(D)** or IFN-λ1 and IFN-λ4 mRNA **(E)** were measured by qRT-PCR; GAPDH expression was measured as a housepkeeping control. Bars indicate ΔΔCt values, normalized according to *gapdh* values and mock results. Data are represented as mean ± *SD* of three independent experiments. #*p* < 0.05 in relation to ZIKV_PE243_.

We have previously demonstrated that dengue virus stimulated HBMEC to produce increased levels of chemokines and proinflammatory cytokines, which might contribute to the inflammatory response observed during the disease (da Conceição et al., 2013). Here, we evaluated whether ZIKV was able to induce the same activation pattern. Supernatants of HBMECs infected with ZIKV_PE243_ were harvested at different time points and levels of IL-6, IL-8, and CCL5 were measured by ELISA. We observed a significant increase in secretion of IL-6 and CCL5, but not IL-8, at 72 hpi, in comparison to mock treated cells (Figure 3A). To investigate whether HBMECs activation would be a unique feature of the Brazilian strains, we compared cytokine secretion induced by ZIKV_PE243_ or ZIKV_MR766_ by 27-plex multiplex analysis (Table 1). The cytokines that showed significant increased levels were confirmed by ELISA in additional experiments. ZIKV_PE243_ and ZIKV_MR766_ stimulated the secretion of IL-6 and CCL5 (Figures 3B,C), whereas CXCL10 secretion was not detected in any situation (data not shown). These data suggest that both viruses activated HBMECs, what may equally contribute to attraction of leukocytes, especially lymphocytes to BBB.

**Table 1:** Comparison of Inflammatory cytokines, chemokines, and growth factors levels in supernatant from human brain microvascular endothelial cell line (HBMEC) after infection with Brazilian and African ZIKV strains (ZIKV_PE243_ and ZIKV_MR766_).

	**Control**	**ZIKV**_**PE243**_ **(MOI 0.1)**		**ZIKV**_**MR766**_ **(MOI 0.1)**	
	**pg/mL**	**pg/mL**	**Fold change**	**pg/mL**	**Fold change**
**IMFLAMMATORY CYTOKINES**					
IL1β	1.11	1.56	1.41	0.76	0.68
IL1ra	28.13	52.9	1.88	52.9	1.88
IL2	–	–	–	–	–
IL4	5.35	10.74	2.01	7.77	1.45
IL5	–	–	–	0.52	–
IL6	356.83	929.23	2.60	469.71	1.32
IL9	–	2.09	–	4.76	–
IL10	12.93	13.33	1.03	37.04	2.86
IL12	62.97	68.49	1.09	197.02	3.13
IL13	1.04	1.15	1.11	2.91	2.80
IL15	–	1.49	–	0.37	–
IL17a	94.98	113.23	1.19	11.4	0.12
IFNγ	46.27	72.54	1.57	90.71	1.96
TNFα	7.04	13.27	1.88	10.16	1.44
**CHEMOKINES**					
Eotaxin	36.45	64.74	1.78	50.08	1.37
IL8	225.15	674.93	3.00	583.62	2.59
CXCL10	54.27	71.46	1.32	71.46	1.32
CCL2	539.54	736.19	1.36	640.37	1.19
CCL3	1.71	1.95	1.14	0.83	0.49
CCL4	0.56	0.91	1.63	0.31	0.55
CCL5	6.50	16.03	2.47	27.63	4.25
**GROWTH FACTORS**					
Basic FGF	97.97	116.48	1.19	21.2	0.22
G-CSF	3.29	13.04	3.96	6.53	1.98
GM-CSF	–	–	–	–	–
IL7	10.43	11.35	1.09	21.36	2.05
PDGF-BB	137.21	364.5	2.66	149.3	1.09
VEGF	1111.64	1236.97	1.11	5505.51	4.95

Expression of type I and type III interferons were also evaluated in infected cells by qRT-PCR. We observed that both virus strains stimulated IFN-β and IFN-λ1 production; however, ZIKV_MR766_ promoted earlier and higher levels of IFN expression (Figures 3D,E). In addition, only ZIKV_MR766_ produced IFN-λ4, although at low levels (Figure 3E). These data suggest that although ZIKV_MR766_ induces increased CPE, virus replication was also associated to enhanced type I and III IFNs, what might contribute to the control of virus dissemination and endothelial lesion *in vivo*.

### ZIKV infection does not induce enhanced permeability of the endothelial cell monolayer

**Figure 4:**
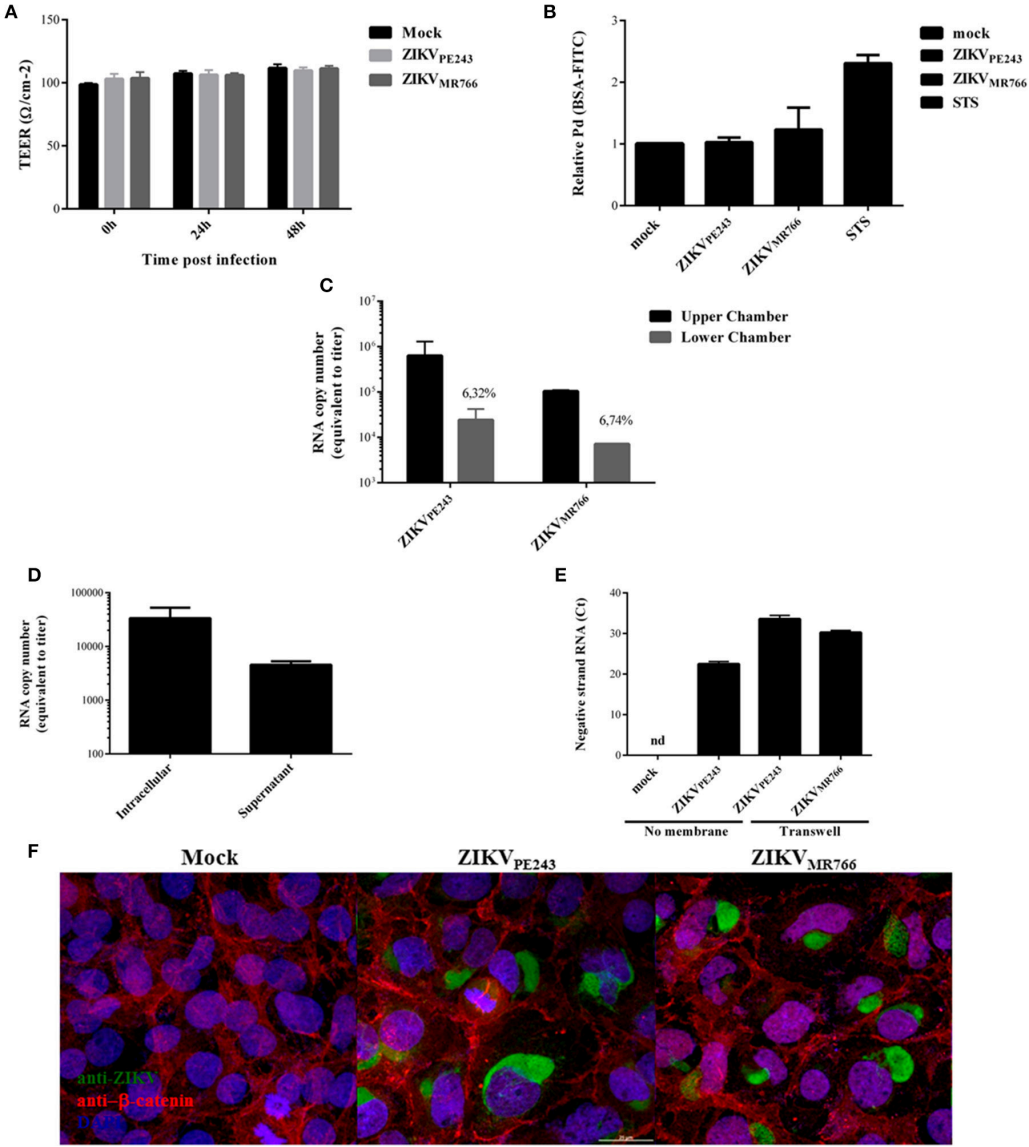
ZIKV crosses endothelial cell monolayer, without increasing permeability. **(A)** HBMECs were cultured onto transwell plates and the cells were mock-treated or infected with ZIKV_PE243_ or ZIKV_MR766_. TEER was measured at 24 and 48 h post infection. **(B)** HBMECs were infected as in **(A)**; as controls, the cells were cultured with staurosporin (STS). After 72 hpi, cells were incubated with FITC-BSA for 30 min, the amount of extravasated albumin was measured by spectrophotometry, and the permeability coefficient (Pd) was calculated and normalized in relation to cells cultured in medium only. **(C)** Virus RNA was measured in the luminal (upper) and abluminal (lower) chambers of the transwell plates by qRT-PCR. Insert numbers indicate the percentage of RNA copies in relation to the corresponding upper chamber. **(D)** Conditioned media harvested from the lower transwell chamber of ZIKV_PE243_ infected cells were inoculated into Vero cells. After 48 h, virus RNA present in the cell lysates and supernatant from Vero cells were measured by qRT-PCR. **(E)** HBMECs and Vero cells were cultured in the upper and lower chamber of a transwell plates, respectively. HBMECs were infected from the apical side, as described. After 72 hpi, Vero cells were harvested, and ZIKV negative strand RNA was measured by qRT-PCR. As positive and negative controls, Vero cells were mock-treated or directly infected with ZIKV_PE243_ (no membrane); nd, not detected. **(F)** HBMECs were cultured as in **(A)**. After 48 hpi, the cells were stained with anti-Flavivirus (4G2 antibody), followed with anti-mouse IgG-AlexaFluor488; and with anti-β-catenin, followed by anti-rabbit IgG-AlexaFluor 594; and with DAPI. ZIKV infection and β-catenin expression were then analyzed by immunofluorescence. Data are represented as mean ± *SD* of four independent experiments.

We finally analyzed whether ZIKV would alter BBB permeability. Cells were seed onto transwell inserts and infected with ZIKV_MR766_ or ZIKV_PE243_, with a MOI of 1. Mock supernatants and staurosporin (STS) were used as negative and positive controls, respectively. Transendothelial electrical resistance (TEER) was measured for 48 h and no significant differences were observed between ZIKV-infected and mock-treated cells (Figure 4A). After 72 hpi, the supernatants were harvested, cells were incubated with FITC-conjugated albumin for 30 min, and the fluorescence intensity was measured in the abluminal chamber. The permeability coefficient (Pd) was calculated and normalized in relation to cells cultures in culture medium only. We did not observe any significant difference in the levels of albumin extravasation at this time point, in comparison to mock-treated cells (Figure 4B).

Interestingly, in spite of the maintenance of endothelial monolayer integrity, virus RNA could be detected in the lower chamber of the transwell system, suggesting that ZIKV may cross BBB through basolateral virus release, transcytosis or paracytosis (Figure 4C). Conditioned medium obtained from the lower chamber of the transwell system was inoculated in ZIKV-susceptible Vero cells and, after 48 hpi, ZIKV RNA was measured in Vero cell lysates and supernatants. Intracellular and supernatant RNA virus were detected in the cultures, indicating that extravasated ZIKV was able to productively infect other cells (Figure 4D). To confirm the infectivity of the viruses crossing the monolayer, Vero cells were seeded in the lower chamber of the transwell system and the presence of virus negative strand RNA was accessed by qRT-PCR. As a negative and positive control, Vero cells were mock treated or directly infected with ZIKV. Virus negative strand RNA was detected in the Vero cell lysates, confirming that the virus crossed the HBMEC barrier, and replicated into the cells in the lower chamber (Figure 4E). These results confirm that ZIKV may cross endothelial cell monolayer without increasing permeability. To further evaluate endothelial monolayer integrity in the cultures, cells were stained with anti-flavivirus 4G2 antibody and anti-β-catenin. Immunofluorescence analysis did not indicate any cell junction disorganization in ZIKV-infected cultures, in comparison to mock-treated cells (Figure 4F).

**Figure 5:**
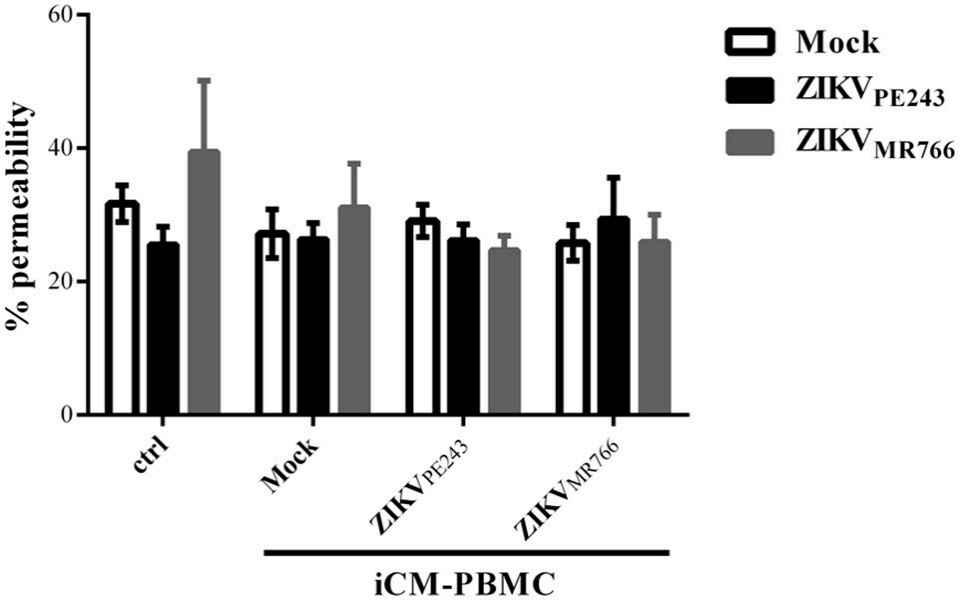
Supernatants from ZIKV-infected PBMC did not induce permeability of ZIKV-infected HBMEC. HBMECs were mock treated or infected with ZIKV_PE243_ or ZIKV_MR766_, in the presence of absence of 50% conditioned inactivated medium obtained from PBMC cultured with mock or infected with ZIKV_PE243_ or ZIKV_MR766_ (iCM-PBMC). Data are represented as mean ± *SD* of five independent experiments.

We then asked whether inflammatory mediators produced by blood cells would be able to affect the permeability of ZIKV-infected HBMECs. PBMC were infected with either ZIKV_PE243_ or ZIKV_MR766_ for 72 h. Then, supernatants were harvested, and virus infectious particles were inactivated by U.V. radiation and used as PBMC conditioned medium (iCM-PBMC). HBMECs were mock treated or infected with ZIKV_PE243_ or ZIKV_MR766_, in the presence of absence of 50% the indicated iCM-PBMC. After 72 hpi, albumin extravasation was measured, as described before. Surprisingly, neither the supernatant obtained from ZIKV_MR766_ nor ZIKV_PE243_-infected cells were able to induce HBMECs permeability in any tested situation (Figure 5). These data indicate that rupture of BBB integrity is not the main pathway stimulated by ZIKV to invade the CNS.

### ZIKV extravasation through BBB may occur through endocytosis/exocytosis replication pathway or through transcytosis

**Figure 6:**
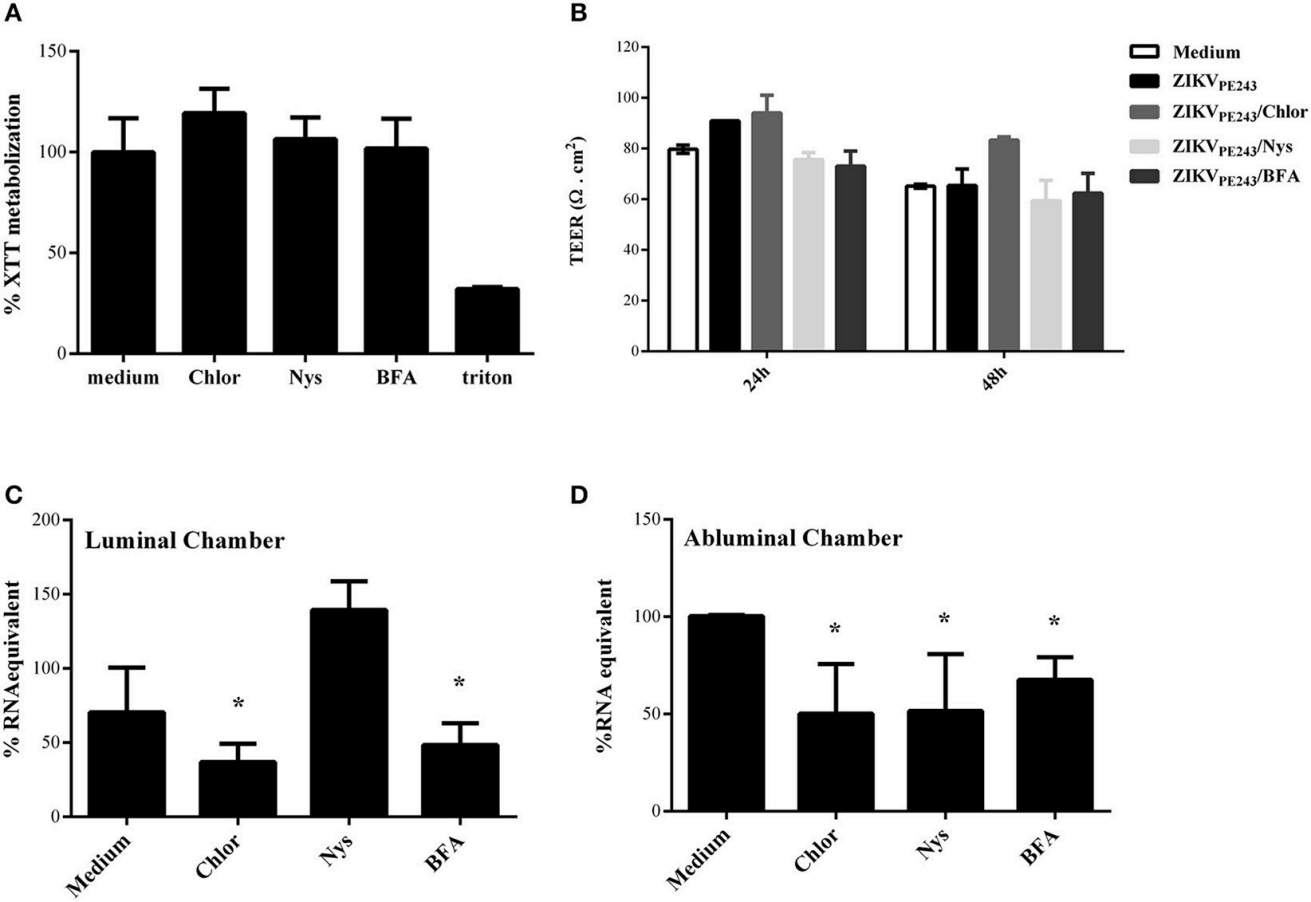
ZIKV extravasation depends on viral replication and basolateral release or transcytosis. **(A)** HBMECs were cultured with chloroquine (Chlor), nystatin (Nys), or brefeldin a (BFA) for 48 h, except for BFA, which was added in the last 10 h culture. Then, cell viability was analyzed by XTT assay. Cells were also cultured with culture medium only or with triton, as negative and positive controls, respectively. **(B–D)** HBMECs were cultured onto transwell systems and then infected with ZIKV_PE243_, from the apical side. The infected cells were treated or not with Chlor, Nys, or BFA and cultured for 48 h. **(B)** TEER was measured at 24 and 48 hpi, **(C,D)** After 48 hpi, the supernatants from the upper (luminal) **(C)** and lower (abluminal) **(D)** chambers were harvested and virus RNA was measured by qRT-PCR. The data indicate the percentage of virus RNA in relation to cells infected in the absence of inhibitors. Data represents the average of three independent experiments; ^*^p < 0.05.

Since virus extravasation was not related to cell death-mediated monolayer disruption, we investigated whether it was dependent on virus replication or transcytosis, by treating ZIKV-infected HBMECs with different pharmacological inhibitors of these pathways. Chloroquine is a weak base able to raise the pH of acidic compartments, which was previously shown to inhibit ZIKV-replication (Delvecchio et al., 2016); nystatin was reported to inhibit virus-induced transcytosis through caveola-mediated pathways (Harmon et al., 2012; Tugizov et al., 2013; Chanthick et al., 2016); brefeldin A (BFA) modulates vesicle traffic from endoplasmic reticulum to Golgi, leading to exocytosis inhibition (Fujiwara et al., 1988). Initially, we analyzed if the drugs would affect cell survival themselves, and none of them were cytotoxic at the concentrations used (Figure 6A). To investigate whether this drugs would affect virus extravasation, cells were cultured onto transwell plates, infected, and treated with them. HBMEC TEER was not altered by any drug during 48 h culture, confirming our previous data that ZIKV does not disturb cell resistance and demonstrating that the drugs did not induce cell permeability themselves (Figure 6B).

Finally, we investigated the effect of each drug in ZIKV replication and extravasation through HBMECs monolayers. Cells were infected with ZIKV_PE243_, from the apical side, and treated with chloroquine, nystatin or brefelding A. The medium obtained from the luminal (upper) and abluminal (lower) chambers were harvested after 48 hpi, and virus RNA in each compartment was measured by qRT-PCR (Figures 6C,D). As expected, analysis of apical release of virus RNA demonstrated that chloroquine and BFA inhibited around 50% ZIKV infection. On the other hand, addition of nystatin did not decrease virus RNA release (Figure 6C). Treatment of ZIKV-infected HBMEC with chloroquine or BFA also resulted in around 50 and 65% decrease of virus RNA in the abluminal transwell chamber, respectively, indicating that virus replication and exocytosis or basolateral release were necessary for extravasation (Figure 6D). Interestingly, despite having no effect in virus replication and apical release, addition of nystatin to the cultures also decreased virus extravasation by around 50% (Figure 6D), suggesting that caveola-mediated transcytosis and/or basolateral release is an important pathway for ZIKV extravasation through brain endothelial cells.

### ZIKV reaches the central nervous system without disrupting the blood brain barrier *in vivo*

**Figure 7:**
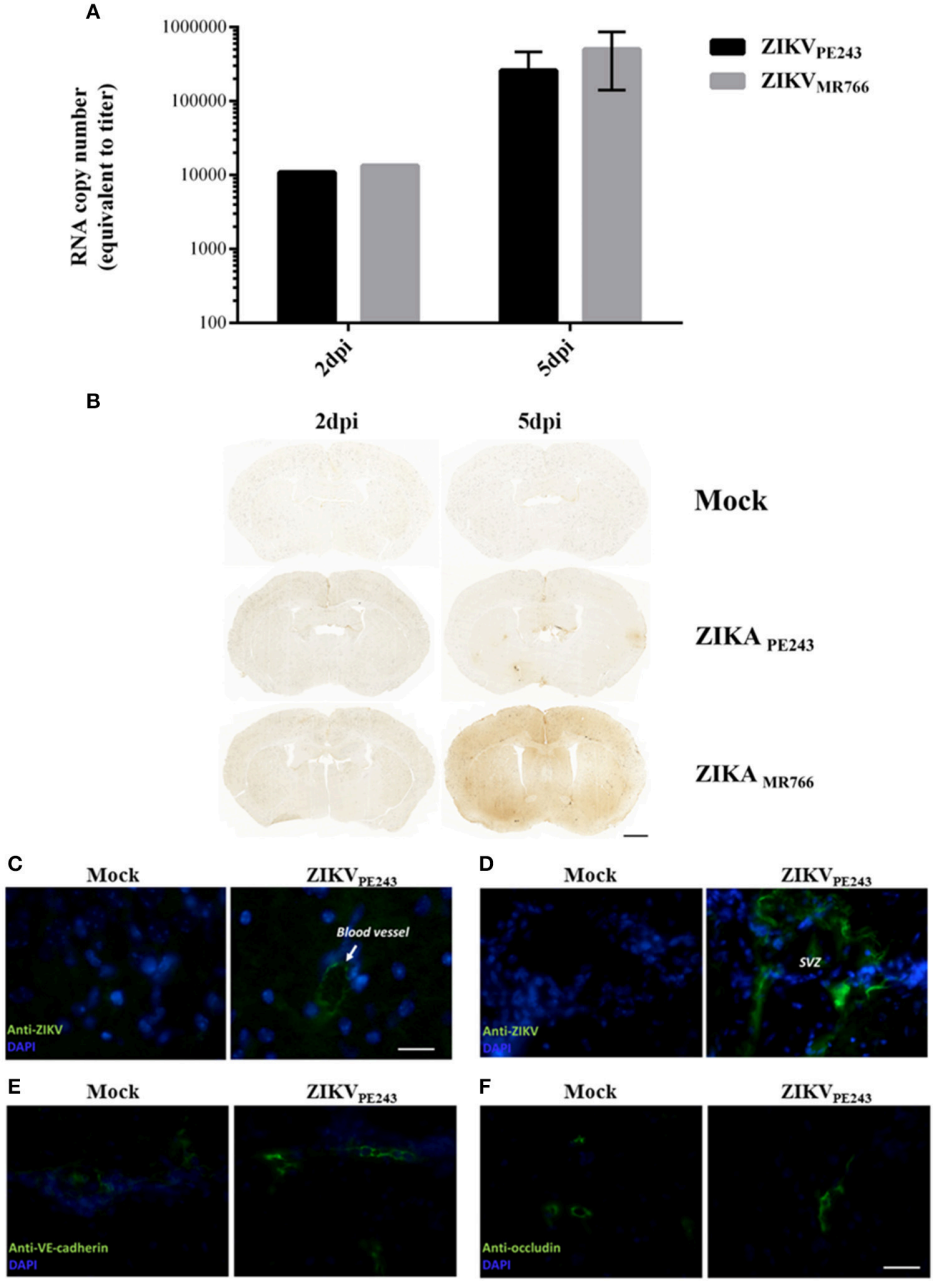
ZIKV reaches mouse brains without disrupting BBB. A129 mice were mock-inoculated or infected with ZIKV_PE243_ or ZIKV_MR766_ (2 × 10^5^ PFU) by i.v. route. **(A)** Virus RNA in the brains obtained from 2 or 5 days infected mice were measured by qRT-PCR. The values indicates the average of RNA copy numbers of four individual mouse infected with the respective viral strain. **(B)** Photomicrographs showing the pattern of immunoglobulin G staining in the brain of young A129 mice infected with ZIKV_MR766_ or ZIKV_PE243_, at 2 or 5 days post infection (dpi). Mice from the control group received an intravenous injection of saline. Scale bar: 1,000 μm. **(C–F)** After 5dpi, mice were transcardially perfused, the brains were harvested, and immunohistochemistry analyses were performed as described. Brain sections were incubated with mouse antibodies anti-4G2 antibody **(C,D)**, anti-VE-cadherin **(E)**, or anti-occludin **(F)**, followed by incubation with AlexaFluor 488-conjugated anti-mouse IgG. Expression of virus E protein and adherens and tight junction were then analyzed using a Zeiss Axio Observer Z1 microscope equipped with an Apotome module.

To confirm that ZIKV was able to reach the brain without disrupting BBB, we evaluated BBB integrity in a mouse experimental model. For this purpose, we infected IFNAR-deficient A129 mice, which was previously demonstrated to be susceptible to ZIKV infection (Dowall et al., 2016; Lazear et al., 2016; Rossi et al., 2016). Mice were intravenously inoculated with ZIKV_PE243_ or ZIKV_MR766_ (2 × 10^5^ PFU). After 2 and 5 days p.i., virus RNA was quantified in the mice brains by qRT-PCR and BBB integrity was accessed by Evans Blue extravasation assay and measurement of endogenous IgG in the brains. ZIKV genome was highly detected in all the brains investigated, from mice infected with either ZIKV_PE243_ or ZIKV_MR766_, at 2 and 5 dpi (Figure 7A). BBB integrity was initially analyzed by inoculating Evans blue dye (EB), i.v., after 2 and 5 dpi. After 1 h, the brains were harvested and the amount of EB per mg tissue was measured. We could not detect a significant amount of EB in the brains obtained from ZIKV-infected mice, in comparison to mock-treated ones (Supplementary Figure 2). Since recent studies have questioned the sensibility of this method (Saunders et al., 2015), we performed another set of experiments, in which the presence of endogenous IgG was histologically evaluated in the brain sections. Corroborating with the previous data, no staining could be detected at 2 dpi in any of the experimental groups (Figure 7B). At 5 dpi, however, a diffuse staining pattern was observed in 1 out of 3 animals infected with ZIKV_MR766_ (Figure 7B). In addition, small, focal areas of immunoglobulin G staining were detected in another animal infected with ZIKV_MR766_ and in 3 out of 4 animals infected with ZIKV_PE243_. No staining for immunoglobulin G was seen in the brain of control mice. These data supports the hypothesis that ZIKV is able to reach the CNS, without disrupting the BBB; however, blood brain barrier disruption may be a delayed event following ZIKV infection in young A129 mice.

Immunostaining for ZIKV envelope protein in the brain sections revealed robust staining in blood vessels (Figure 7C) and in the cells of plexus choroid of subventricular zone (SVZ) (Figure 7D), indicating that brain endothelial cells are actually infected by ZIKV. We also performed immunostaining of VE-cadherin and occludin proteins and we found no changes in the expression profile of these adherens and tight junctions markers in the brains of ZIKV-infected mice (Figures 7E,F), further suggesting that BBB was not remarkably affected.

Taken together, our data indicate that ZIKV productively infects HBMECs, and induces cellular activation. However, infection did not induce BBB permeability, *in vitro* nor *in vivo*, suggesting that ZIKV is able to reach the CNS without disrupting the BBB and that the invasion of CNS by ZIKV might be associated to transinfection or transcytosis from the endothelial cells of the BBB. However, the persistent CPE caused by the presence of ZIKV_MR766_ and/or the subsequent inflammation triggered by the replication either virus strain in the brains may induce a slight disruption of BBB at later time points.

## Discussion

In the present work, we demonstrated that ZIKV infection of brain endothelial cells result in cellular activation and release of infectious virus particles, with no increase of endothelial monolayer permeability *in vitro* and no significant disruption of BBB *in vivo*.

ZIKV infection has been associated to alteration of CNS, including microcephaly and other neurological abnormalities after congenital infection, and meningoencephalitis in adults (Calvet et al., 2016; Carteaux et al., 2016; Cugola et al., 2016; Driggers et al., 2016; Martines et al., 2016; Miranda-Filho et al., 2016; Mlakar et al., 2016). Virus was detected in the brains from microcephaly and meningoencephalitis cases, indicating that ZIKV might cross the BBB and infect CNS cells (Cugola et al., 2016; Driggers et al., 2016; Martines et al., 2016; Mlakar et al., 2016). However, the mechanism by which the virus reaches the brain has not been addressed yet.

Our results demonstrated that both Brazilian and African ZIKV strains efficiently infected HBMECs and induced the secretion of type I and III IFNs and inflammatory cytokines. Infection of HBMECs by a Puerto Rican ZIKV strain (from Asian lineage) was recently demonstrated and the secretion of chemokines and interferons were also observed (Mladinich et al., 2017). Also, that article described that HBMECs became resistant to IFN effects and to virus-induced cell death, and that ZIKV infection did not result in increased permeability of HBMECs (Mladinich et al., 2017). The Brazilian lineages used in this study were isolated from patients with mild disease and both isolates were phylogenetically related to the Asian lineage of ZIKV. Brazilian ZIKV lineages behaved quite similar regarding the infection rate and cell death induction, showing less CPE than ZIKV_MR766_, in accordance with the previous data. These data suggest that the differences in the sequences of ZIKV lineages might affect virus-induced cytotoxicity, although we cannot discard that ZIKV_MR766_ fitness might be related to higher passage numbers of this reference strain, in comparison to the Brazilian strains used in this study, which were under eight passages in culture. Other studies suggested that ZIKV-BR induced a higher cytopathogenic effect than ZIKV-AFR, but those were conducted in human neuron derived from pluripotent cells (NPC) and in human neurospheres (Cugola et al., 2016). Still, these findings need further investigation and may indicate that a higher CPE induction on brain endothelial cells might not be related to increased cases of microcephaly and other CNS alterations observed during infection with Brazilian ZIKV. In fact, the increased cell death induced by ZIKV_MR766_ might contribute to limit virus dissemination and benefit immune control. Interestingly, we observed that, although both ZIKV_PE243_ and ZIKV_MR766_ induced IFN-β and IFN–λ expression, the African strain induced a higher and earlier IFN production. Similar findings have been described in an immunodeficient mouse experimental models, in which infection with African strains were more aggressive than with Asian ZIKV strains (Tripathi et al., 2017). These findings suggest that despite the supposed higher virulence of African ZIKV isolates, the earlier and increased production of type I IFN may contribute to controlling of virus dissemination *in vivo*. However, virus control is not apparent in mouse models, which does not respond to IFN, such as IFNAR or Stat2 deficient mice.

We also observed that both virus strains induced HBMEC activation, with production of IL-6 and CCL5 at similar levels, which might contribute to the recruitment and activation of inflammatory cells. These data support the previous results showing increased expression of inflammatory mediators, particularly CCL5, in HBMECs infected by ZIKV (PRVABC59 strain) (Mladinich et al., 2017). Analysis of inflammatory mediators in the cerebrospinal fluid (CSF) of stillborns and in the brains of mouse experimental models also demonstrated increased levels of IL-6, CCL5, and type I IFNs (Galliez et al., 2016; Tripathi et al., 2017). Therefore, brain endothelial cell activation might contribute to the inflammatory milieu detected upon ZIKV infection. Also, analysis of stillborns tissues revealed the presence of a few inflammatory infiltrates circumventing the neurons (Mlakar et al., 2016), what might result from the production of chemokines by local infected cells. However, the ability of leukocytes to cross the BBB during infection still needs to be investigated.

Several neurotropic viruses access the CNS as free virions or cell-associated from the bloodstream. Those may use different pathways to gain access to CNS, including direct transport from peripheral nerves, and transcytosis or transinfection. *In vitro* infection of HBMECs and *in vivo* infection of A129 mice with ZIKV_PE243_ did not result in significant BBB disruption, although virus could be clearly detected in the brains. It is in agreement with the fact that ZIKV from Asian lineages did not result in a marked cytophatic effect of HBMECs *in vitro*, and that apical or basolateral infection of HBMECs with ZIKV induced virus release without evidences of disruption of monolayer integrity, which was observed here and in a previous study (Mladinich et al., 2017). Our data also demonstrated that despite the downregulation in metabolism and cell death induced by ZIKV_MR766_, no major disruption of endothelial monolayer occurred *in vitro*, and BBB disruption was not essential for the virus to reach the brain. However, at late time points after infections with ZIKV_MR766_, hemorrhagic points could be detected in the brain sections, suggesting that the prolonged lesion induced by the presence of this virus in the brains may contribute to a late disruption of the BBB. These data also suggest that other mechanisms, but not disruption of endothelial layers in the BBB might be involved in CNS invasion by the virus. Indeed, treatment of infected HBMECs with drugs that inhibited virus replication or transcytosis significantly impaired virus extravasation through cells monolayer in a transwell system.

Chloroquine was previously demonstrated to inhibit cellular infection (Delvecchio et al., 2016), possibly due to increased endosomal pH and prevention of virus uncoating. BFA is an exocytosis inhibitor, and might, therefore, block Flavivirus budding. In fact, HBMEC treatment with both drugs inhibited virus RNA release and extravasation of infectious virus in a transwell system, indicating that active replication was necessary to ZIKV-crossing through BBB. On the other hand, addition of nystatin to the cultures, despite not affecting virus replication levels nor apical RNA release, significantly inhibited virus crossing through the transwell. These data suggest that caveola-mediate traffic might not be essential for ZIKV replication, but may participate in virus basolateral release, or that transcytosis pathway may also take part in the process of virus extravasation.

Neurotropic viruses, such as poliovirus, rabhdovirus, and human herpes virus 1 and 2 were reported to reach the CNS through peripheral nerves (Finke and Conzelmann, 2005; Racaniello, 2006; Diefenbach et al., 2008; Luethy et al., 2016). Axonal transport and dissemination through peripheral nerves might also happen during Flavivirus infection, and it was described that a direct inoculation of WNV in the sciatic nerve of a hamster experimental model promoted limp paralysis (Samuel et al., 2007). However, Flaviviruses usually reach the CNS from the blood. Mouse experimental models demonstrated that YFV17D access the brains mostly hematogenously (Luethy et al., 2016). In addition, WNV were also reported to cross and disrupt BBB, in a way dependent on systemic inflammatory responses (Wang et al., 2004), although a report had demonstrated that BBB disruption was not essential for lethal WNV infection (Morrey et al., 2008). In a mouse model using adapted neurovirulent DENV strain, BBB disruption was observed, with infection of neurons, microglial and endothelial cells, associated to leukocyte infiltration (Velandia-Romero et al., 2012). On the other hand, JEV was reported to infect brain astrocytes, leading to cell activation and production of inflammatory cytokines, VEGF, and metalloproteinases, which then, seemed to affect neighbor endothelial cells, and to disrupt BBB (Chang et al., 2015). In fact, direct effect of JEV in electrical resistance or permeability of cultured endothelial cells were barely observed (Chen et al., 2014). Astrocytes were also demonstrated to be preferentially infected by ZIKV in an *ex vivo* model of organotypic cultures from primary human brain tissue (Retallack et al., 2016). In addition, another study demonstrated that THP-1 cells infected with ZIKV could cross endothelial cells in a transwell system, leading to infection of astrocytes cultured in the basolateral chamber of the transwell (Bramley et al., 2017). Given that astrocytes directly interact with endothelial cells in the brains and that such interaction might be relevant for the formation and organization of the BBB (Janzer and Raff, 1987), infection of these cells after ZIKV crossing through the endothelial layer may contribute to subsequent inflammation and alteration of the barrier.

ZIKV infection of peripheral neurons has never been addressed and dissemination through peripheral nerves might not be the main pathway of virus spreading to CNS, although it cannot be discharged. It is possible then that virus hematogenous dissemination allow virus access to BBB and infection of endothelial cells, promoting virus transcytosis or basolateral release, and infection of brain cells. Viral access to CNS by transcytosis has been demonstrated in other virus infections models, including WNV and JEV and may be a common pathway through which flavivirus reach CNS (Liou and Hsu, 1998; Verma et al., 2009; Dohgu et al., 2012).

Finally, infection of endothelial cells has been demonstrated in other tissues and organs crossed by ZIKV and associated to Zika syndrome, such as placenta, eye, and brain (Noronha et al., 2016; Roach and Alcendor, 2017; Singh et al., 2017; Vermillion et al., 2017). Regarding retinal endothelial cells, increased levels of antiviral and pro-inflammatory cytokine expression was also observed, including IFN-β, IL-6, and CCL5 (Roach and Alcendor, 2017; Singh et al., 2017), similar to what we observed in HBMECs. Activation of endothelial cells may also contribute to alteration of BBB integrity, which was detected at later time points after infection, especially upon infection with ZIKV_MR766_. In fact, we demonstrated that the virus is able to cross the endothelial barrier without disrupting it, which could be an initial step of virus release to CNS, allowing the infection and activation of other cells types and contributing to the amplification of the lesion, as discussed.

In summary, we demonstrated that ZIKV efficiently infect HBMECs, resulting in release of infectious virus particles, what may contribute to virus access to CNS. HBMECs were also activated by the infection, producing inflammatory cytokines and chemokines, which can be relevant for the recruitment and activation of leukocytes and amplification of the inflammatory response *in vivo*. Those inflammatory mediators might also affect survival, activation and infection of other cells present in the brain. Importantly, endothelial cells are part of the two main barriers to be crossed by ZIKV to cause severe disease—placental and BBB. Fetal endothelium was also demonstrated to be infected by ZIKV in mouse experimental models (Miner et al., 2016), but the direct role of this infection for placental extravasation or fetal vascular alteration was not investigated yet. Therefore, the study of infection of endothelial cells by ZIKV might contribute in further understanding of the mechanisms of virus dissemination and congenital disease, including CNS alterations.

## Author contributions

LBA: conceived and supervised the experiments and data analysis. MP, LM, YM: performed and analyzed the *in vitro* experiments. MP, LM, and LBA: wrote the manuscript draft. SC, CL, MR, and MN: developed the methodology for virus production and mouse infection. MP, SC, CL, PF, MB, CF and HP, and PP-C: performed and supervised the *in vivo* experiments. PF, LC, PP-C, FL, and CF: performed image analysis of *in vitro* and *in vivo* assays. PS, PP, AT, and RSA: performed the analyses of negative strand RNA expression and cytokine production by multiplex. MB, AT, MN, RSA, and LBA were responsible for resources, funding acquisition, data interpretation, and write-review and editing. All authors reviewed the manuscript.

### Conflict of interest statement

The authors declare that the research was conducted in the absence of any commercial or financial relationships that could be construed as a potential conflict of interest. The reviewer KS and handling Editor declared their shared affiliation.

## Supplementary material

Supplementary Figure 1 ZIKV_PE243_ does not induce severe CPE in HBMECs. **(A)** HBMECs were mock-treated or cultured with ZIKV_PE243_ or ZIKV_MR766_, produced in C6/36 cells. Cells were stained with Live/Dead kit and were evaluated by flow cytometry, at the indicated time points. Plots demonstrates a representative experiment of two independent experiments. **(B)** HBMECs were mock-treated (ctrl-) or infected with ZIKV_PE243_ or ZIKV_MR766_. After 48 hpi, cultures were stained with PI (left panel) or TUNEL (right panel) and analyzed by fluorescence microscopy. Triton X-100 and staurosporin were used as positive controls (ctrl+) for PI and TUNEL staining, respectively. **(C,D)** HBMECs **(C)** or Vero cells **(D)** were mock treated or infected with ZIKV_PE243_ or ZIKV_MR766._ After 48 hpi, cells were tripsinized and counted using trypan blue exclusion dye. Data are represented as mean ± *SD* of two independent experiments.

Supplementary Figure 2 ZIKV reaches mouse brains without disrupting BBB. A129 mice were mock-inoculated or infected with ZIKV_PE243_ or ZIKV_MR766_ (2 × 10^5^ PFU) by i.v. route. As a positive control of BBB disruption, some mice were inoculated with Plasmodium berguei ANKA, a model of cerebral malaria. After 2 or 5 days post infection, mice were i.v. injected with 0.5% Evans blue solution (EB); as a control some mice were injected with PBS (ctrl-). After 1 h, the brains were removed; liver, kidneys and spleen were also removed as controls, and all the organs were photographed for visualization of Evans Blue staining **(A)**. The amount of Evans blue was measured by optical spectroscopy, and the concentration of EB/mg tissue was calculated **(B)**.
