# Supplementary figures and images for: Zika Virus Infects, Activates, and Crosses Brain Microvascular Endothelial Cells, without Barrier Disruption
Source: Front Microbiol. 2017 Dec 22;8:2557. doi: 10.3389/fmicb.2017.02557 (PMC5743735; doi:10.3389/fmicb.2017.02557)

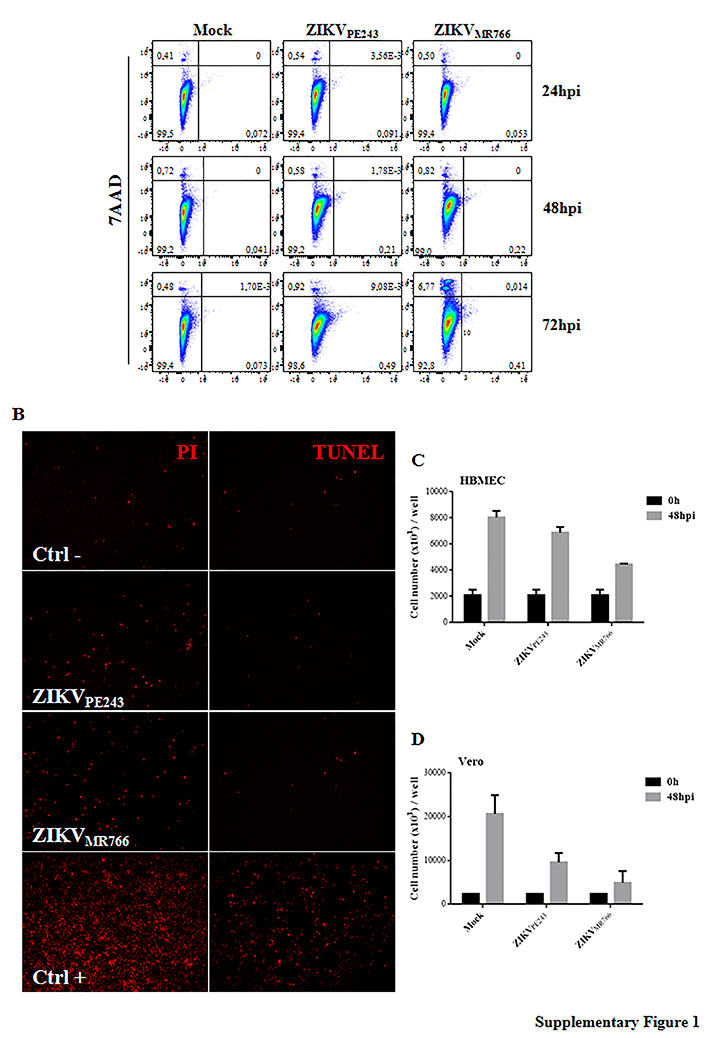

Supplement: Supplementary Figure 1 — ZIKVPE243 does not induce severe CPE in HBMECs. (A) HBMECs were mock-treated or cultured with ZIKVPE243 or ZIKVMR766, produced in C6/36 cells. Cells were stained with Live/Dead kit and were evaluated by flow cytometry, at the indicated time points. Plots demonstrates a representative experiment of two independent experiments. (B) HBMECs were mock-treated (ctrl-) or infected with ZIKVPE243 or ZIKVMR766. After 48 hpi, cultures were stained with PI (left panel) or TUNEL (right panel) and analyzed by fluorescence microscopy. Triton X-100 and staurosporin were used as positive controls (ctrl+) for PI and TUNEL staining, respectively. (C,D) HBMECs (C) or Vero cells (D) were mock treated or infected with ZIKVPE243 or ZIKVMR766. After 48 hpi, cells were tripsinized and counted using trypan blue exclusion dye. Data are represented as mean ± SD of two independent experiments. [file Image1.TIF]

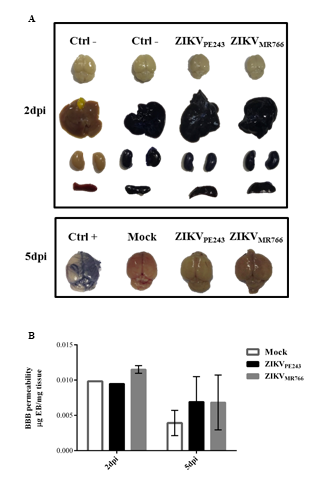

Supplement: Supplementary Figure 2 — ZIKV reaches mouse brains without disrupting BBB. A129 mice were mock-inoculated or infected with ZIKVPE243 or ZIKVMR766 (2 × 10^5^ PFU) by i.v. route. As a positive control of BBB disruption, some mice were inoculated with Plasmodium berguei ANKA, a model of cerebral malaria. After 2 or 5 days post infection, mice were i.v. injected with 0.5% Evans blue solution (EB); as a control some mice were injected with PBS (ctrl-). After 1 h, the brains were removed; liver, kidneys and spleen were also removed as controls, and all the organs were photographed for visualization of Evans Blue staining (A). The amount of Evans blue was measured by optical spectroscopy, and the concentration of EB/mg tissue was calculated (B). [file Image2.tif]
